# Prognostic value of intraoperative neural monitoring of the recurrent laryngeal nerve in thyroid surgery

**DOI:** 10.1007/s00423-016-1441-0

**Published:** 2016-05-03

**Authors:** Małgorzata Stopa, Marcin Barczyński

**Affiliations:** 1 Department of Endocrine Surgery, Third Chair of General Surgery, Jagiellonian University Medical College, Kraków, Poland; 2 Third Chair and Department of General Surgery, Jagiellonian University, Medical College, 37 Prądnicka Street, 31-202 Kraków, Poland

**Keywords:** Thyroid surgery, Intraoperative neuromonitoring, Recurrent laryngeal nerve, Prognostic value, Staged thyroidectomy

## Abstract

**Purpose:**

The diagnostic accuracy of intraoperative recurrent laryngeal nerve (RLN) monitoring (IONM) remains controversial. The aim of this study was to evaluate IONM diagnostic accuracy in prognostication of postoperative nerve function in thyroid surgery.

**Methods:**

This prospective study was conducted in 2011–2013. Five hundred consenting patients qualified for total thyroidectomy with IONM (1000 nerves at risk) using NIM 3.0 Response equipment were included. Laryngoscopy was used to evaluate and follow up RLN injury. The primary outcome was diagnostic accuracy of IONM. The receiver operating characteristics (ROC) were used for evaluation of IONM diagnostic accuracy.

**Results:**

Loss of signal (LOS) occurred in 31 cases, including 25 patients with LOS and corresponding vocal fold paresis found in postoperative laryngoscopy (2.5 %), including 20 (2.0 %) temporary and 5 (0.5 %) permanent nerve lesions. The following diagnostic accuracy values were calculated for the criterion recommended by INMSG (V2 amplitude ≤ 100 μV): sensitivity 92.0 %, specificity 99.3 %, positive predictive value (PPV) 76.7 %, and negative predictive value (NPV) 99.8 %. The ROC curve analysis allowed for calculation of the most optimal criterion in prognostication of postoperative vocal fold paresis, namely, V2 amplitude ≤ 189 μV. For this criterion, PPV was 77.4 %, while NPV was 99.9 %.

**Conclusions:**

Adherence to the standardized protocol recommended by the International Neural Monitoring Study Group allows for optimizing predictive values of IONM in prognostication of postoperative RLN function. Any changes in the cutoff values for the definition of LOS only marginally improve PPV and NPV of IONM and need to be carefully assessed in multicenter studies.

## Introduction

Stimulation techniques allow for certain identification of the recurrent laryngeal nerve (RLN) and the external branch of the superior laryngeal nerve [1–3]. Nevertheless, RLN identification is often hindered due to neck anatomy altered by a large-size goiter, advanced-stage thyroid cancer, or scarring after previous procedures [2, 4]. In such situations, the neuromapping technique may be of high assistance, allowing for nerve localization prior to its visualization through repeated tissue stimulation in the surgical field [2, 4]. To date, the value of the technique has not been unambiguously evaluated in research studies. Neuromapping may also help in evaluating mechanisms underlying RLN injuries, which is closely associated with its diagnostic value in prognostication of postoperative nerve function [2]. The assessment of qualitative (signal preservation or loss) or quantitative parameters (change of signal amplitude and/or latency) of electromyographic recording may be the basis for intraoperative prognostication of nerve function in the postoperative period [1, 3]. Although, to date, publications emphasize a high variability of predictive value of both a positive result (loss of signal), which may be 10–90 %, and a negative result (preservation of normal signal) which may be 70–99 % [5–8], the possibility of assessing preservation of functional nerve integrity by intraoperative neuromonitoring (IONM) represents significant progress as compared to assessing solely anatomical nerve integrity [2]. The issue is particularly important in preventing bilateral RLN injuries, being associated with the problem of staged thyroidectomy consisting in abandoning operating the contralateral side (or limiting the scope of contralateral lobectomy) in case loss of signal (LOS) occurs during resection of the first thyroid lobe [9–11]. Recent studies suggest a possibly improved predictive IONM value based on intraoperative analysis of surface electromyography recording from vocal muscle signal and on employment of the standardized IONM methodology developed by the International Neural Monitoring Study Group (INMSG), with prognostication using intraoperative changes of signal amplitude and latency time following vagal nerve stimulation after ipsilateral thyroid lobe resection and correlating such changes with postoperative RLN dysfunction [2, 8]. The issue is currently a subject of numerous studies. Their results may affect the presently developed algorithm of thyroid surgery tactics employing IONM of the RLN and allowing for performing a meta-analysis of data originating from various centers.

The hypothesis explored in this study was that the use of the standardized approach to IONM as proposed by the INMSG may optimize the predictive values of this method in prognostication of postoperative RLN function. The aim of this study was to validate the diagnostic accuracy of IONM in prognostication of postoperative RLN function in thyroid surgery.

## Methods

### Study design and patients

This was a prospective cohort study of 500 patients (1000 RLNs at risk) who were qualified for total thyroidectomy with IONM and were treated at the Third Department of General Surgery, Jagiellonian University Medical College, Krakow, Poland, in 2011–2013. The patients’ characteristics are presented in Table 1.

**Table 1:** Characteristics of patients

	Patients in the study (*n* = 500)
RLNs at risk, no.	1000
Female/male ratio, no.	452:48
Median age (range), years	58 (18–79)
Preoperative diagnosis, no. (%)	
Non-toxic multinodular goiter	326 (65.2)
Toxic multinodular goiter	64 (12.8)
Graves’ disease	21 (4.2)
Differentiated thyroid cancer	64 (12.8)
Primary thyroid surgery, no. (%)	435 (87.0)
Revision thyroidectomy, no. (%)	65 (13)
Thyroid specimen weight, mean ± SD, g	98.0 ± 50.0
Operating time, mean ± SD, min	102.5 ± 31.8

Data are numbers with percentages in parentheses unless otherwise indicated

*RLN* recurrent laryngeal nerve, *LOS* loss of signal

The inclusion criterion was planned total thyroidectomy with IONM. The exclusion criteria were the surgeon’s decision not to use IONM during the case, preoperative vocal fold paresis, and incomplete clinical data or follow-up information.

The primary outcome measure was diagnostic accuracy of IONM in prognostication of postoperative RLN function after thyroid surgery.

Flexible laryngoscopy was mandatory in all the patients before operation and afterwards, being used to evaluate and follow RLN injury. All the patients provided written informed consent for the storage and use of their data. The protocol of this study was approved by the Institutional Review Board (KBET/76/B/2010). This study was reported in agreement with the Standards for the Reporting of Diagnostic Accuracy Studies (STARD) statement updated in 2015 [12].

### Surgical technique

All the operations were performed under general anesthesia by two experienced endocrine surgeons (MS, MB) with annual volume of thyroid surgery >200 cases, each. The anesthesia protocol included intravenous midazolam premedication; induction with fentanyl, thiopental, and suxamethonium; endotracheal intubation; and sevoflurane maintenance. No other muscle relaxants were used during surgery. A standard cervicotomy was used in all the patients. Visual identification of RLN low in the neck (below the crossing with the inferior thyroid artery) was facilitated by the use of the IONM system employing the nerve mapping technique. Once the nerve was visually identified, repeated stimulations with the IONM monopolar probe served to trace the nerve path in the operative field and test its functional integrity during dissection. In each patient, RLN was exposed and the branches of the superior and inferior thyroid arteries were divided close to the thyroid capsule.

### IONM technique

During this study period, the NIM® 3.0 system (Medtronic, Jacksonville, USA) was used. The NIM® system operated with surface electrodes integrated with an endotracheal tube 7.0–7.5 mm in diameter, which was inserted by an anesthetist between the vocal folds under direct vision during intubation. The standardized technique of IONM RLNs was used, including indirect vagal response evaluation at the beginning and at the end of surgery (IONM = L1 + V1 + R1 + R2 + V2 + L2) according to the recommendations formulated recently by the International Intraoperative Neural Monitoring Study Group [2]. The nerves were stimulated using a monopolar electrode and the interrupted stimulation technique at 1 mA, 100 ms impulse duration, and 4 Hz frequency. In case of the bifurcated RLN nerves, the assessment included post-stimulation response of each nerve branch. Adduction of the vocal folds was detected by the endotracheal tube electromyography and abduction by finger palpation of muscle contraction in the posterior cricoarytenoid (“laryngeal twitch”) [13].

LOS was defined as absence of electromyography (EMG) signal following stimulation of the ipsilateral vagus nerve, EMG signal amplitude below 100 μV following stimulation with 1–2 mA current in dry field, lack of palpable laryngeal twitch, or visible laryngeal movement following stimulation of the ipsilateral vagus nerve [2]. To differentiate between true and false LOS, the INMSG-proposed problem-solving algorithm [2] was employed intraoperatively. In cases intraoperatively recognized as true LOS, the neuromapping technique was used to determine the character of nerve damage (segmental—type I, global—type II) and the localization of the injury site.

IONM assessment was based on the definition by Chan and Lo [6]. The percentage of RLN dysfunctions was calculated per number of RLNs at risk and not per number of patients. Loss of signal after vagal stimulation following thyroid lobe resection (V2) was classified as a positive test result prognosticating ipsilateral vocal cord paresis. The test was interpreted as true positive (TP) when laryngoscopy confirmed ipsilateral vocal cord paresis and false positive (FP) when the mobility of the ipsilateral vocal fold was normal. Preserved normal signal following vagal stimulation after thyroid lobe resection (V2) was classified as a negative result that prognosticated normal postoperative mobility of the ipsilateral vocal fold. The test was interpreted as true negative (TN) when laryngoscopy demonstrated postoperative normal mobility of the ipsilateral vocal fold and as false negative (FN) when ipsilateral vocal fold paresis was seen postoperatively.

### Perioperative management and follow-up

Flexible laryngoscopy by a throat specialist was mandatory before surgery and on day 1 postoperatively. In patients with RLN paresis, an additional examination was scheduled at 1, 2, 4, and 6 months after surgery or until the vocal cord function was recovered. Vocal cord paresis for more than 6 months after the operation was regarded as permanent palsy.

### Statistical analysis

The resultant data were statistically processed using the statistical software MedCalc (version 13, MedCalc Software, Belgium). Assessment of the changeability of the investigated parameters was presented by arithmetic means, median values, standard deviations (SD), minimum and maximum values (min-max), 95 % confidence interval (95 % CI), and percentage of prevalence (%). An inter-group comparison of particular properties was done by means of the *χ* ^2^ test (non-parametric variables) and by the univariate analysis of variance ANOVA (parametric variables). To assess the diagnostic accuracy of IONM, the receiver operating characteristic (ROC) curves were analyzed and the area under curve (AUC) values were compared based on the non-parametric method of DeLong et al. [14]. Thus, the predictive values of the positive and negative results were calculated and the most optimal predictive criterion was identified. The incidence of nerve events was calculated based on the number of nerves at risk. The significance level was accepted at *p* < 0.05.

## Results

**Fig. 1:**
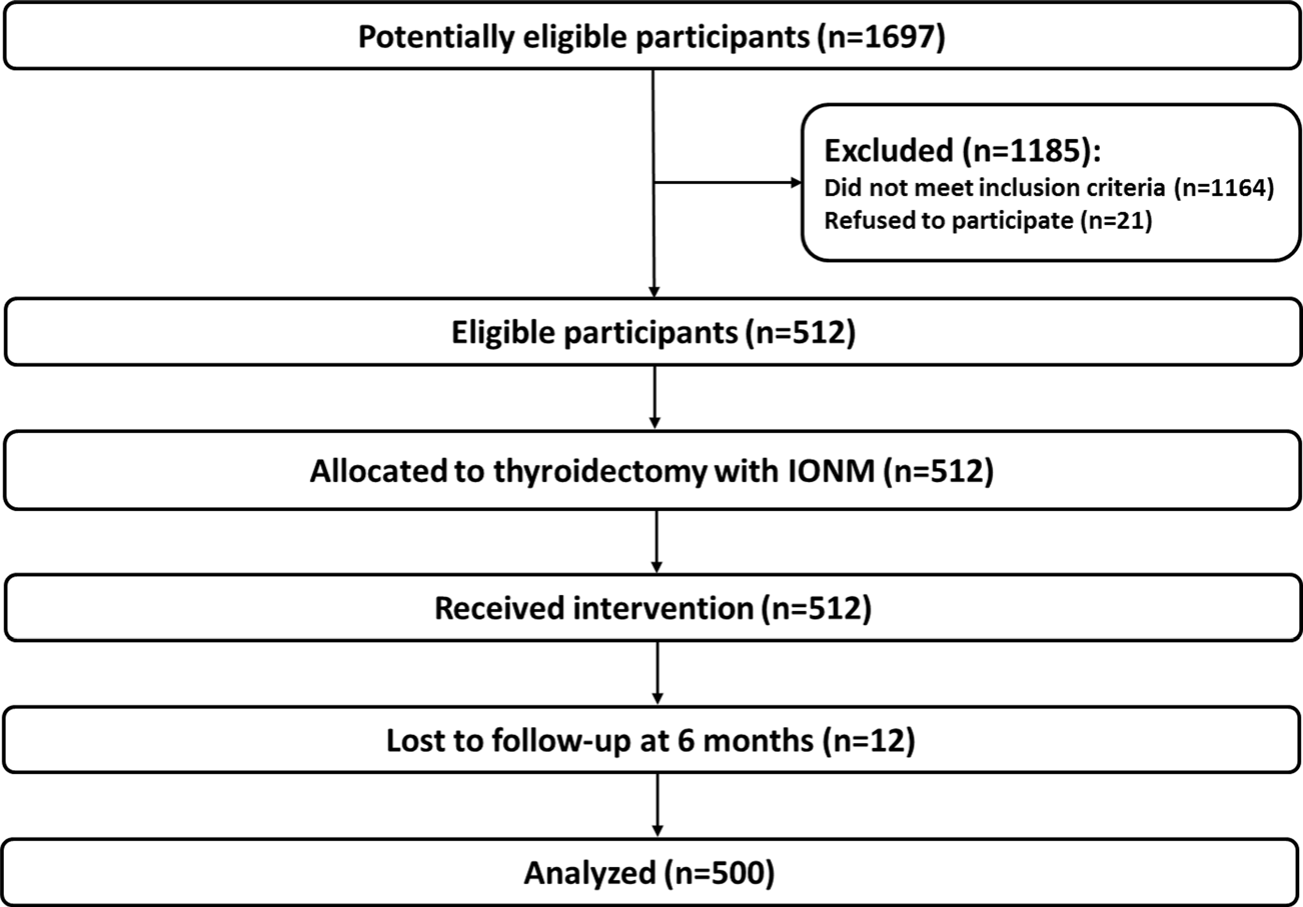
Flow of patients in this study

Of 2034 patients referred for thyroid surgery during the study interval, 337 underwent hemithyroidectomy while 1697 were planned for total thyroidectomy and were potential candidates for the study. One thousand one hundred and sixty-four patients did not meet the inclusion criteria, as there was no plan for utilization of IONM during surgery, while 21 subjects refused to participate, leaving 512 eligible patients of which 12 patients were lost to follow-up at 6 months leaving finally 500 patients (1000 nerves at risk) who were included in the analysis. The flowchart of patients in this study is shown in Fig. 1. There were 452 women and 48 men, with a median age at diagnosis of 58 years (range 18–79).

The clinical characteristics of the patients analyzed in this study are shown in Table 1.

Intraoperative LOS was noted in 31 cases. Of 25 patients with intraoperative LOS and early ipsilateral vocal fold paresis in postoperative laryngoscopy (true positive result, TP), intraoperative RLN neuromapping demonstrated segmental type damage (type I) in 11 cases and global type (type II) in 14 cases. None of the patients showed interruption of anatomical RLN continuity. Anterior RLN stimulation caudally from the site of its entry to the larynx allowed for localizing the LOS site in seven cases at the level of the Berry ligament and in four cases at the level of its crossing the inferior thyroid artery. Of 25 cases (100 %) of early RLN damage, 20 (80 %) were transient and 5 (20 %) permanent.

In 3 of 11 patients with LOS found after resection of the initially dissected thyroid lobe, staged thyroidectomy was done, whereas the remaining 8 patients had one-stage limited contralateral thyroid lobe surgery (Dunhill operation) to minimize the risk of bilateral vocal fold paresis.

### Primary outcome analysis

**Fig. 2:**
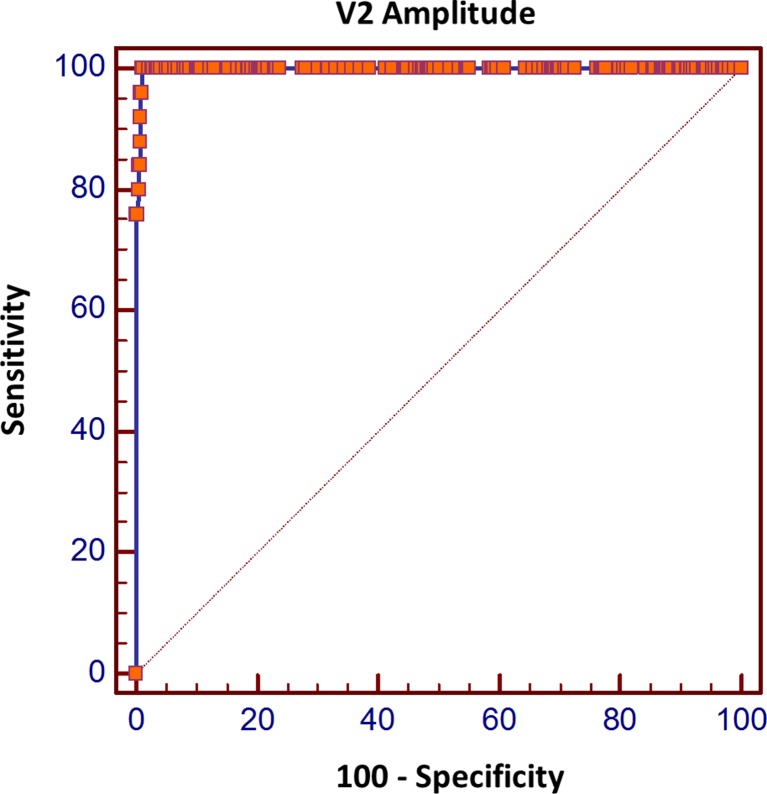
Receiver operating characteristic (ROC) curve showing the diagnostic accuracy of V2 amplitude of EMG following stimulation of the ipsilateral vagus nerve after thyroid lobectomy in prognostication of postoperative function of the ipsilateral vocal fold. Area under curve (AUC) = 0.997 (95 % CI 0.992–1.000, *p* < 0.001)

Using the definition of LOS from the vagus nerve following thyroid lobe resection recommended by INMSG (V2 amplitude <100 μV), in the described group of 500 surgical patients (1000 RLNs at risk) the authors noted 969 (96.9 %) TN, 23 (2.3 %) TP, 2 (0.2 %) FN, and 6 (0.6 %) FP results. Thus, the sensitivity of the criterion was 92.0 % (95 % CI 74.0–99.0 %), specificity 99.3 % (95 % CI 98.5–99.7 %), positive result predictive value 76.7 % (95 % CI 57.3–90.3 %), and negative result predictive value 99.8 % (95 % CI 99.3–100.0 %). The ROC curve analysis allowed for calculating the most optimal criterion in prognostication of postoperative vocal fold paresis as the absolute value of V2 amplitude ≤189 μV, which was characterized by the highest diagnostic accuracy (Fig. 2). For this criterion, positive result predictive value was 77.4 % (95 % CI 58.5–90.6 %) and negative result predictive value 99.9 % (95 % CI 99.0–100.0 %). An additional analysis of the ROC curve that illustrated relative V2 amplitude in relation to initial V1 amplitude allowed for calculating the most optimal criterion in prognostication of postoperative vocal fold paresis as a drop in V2 amplitude below 18.4 % of initial V1 amplitude value (or by more than 81.6 %); the criterion was characterized by positive result predictive value of 76.6 % (95 % CI 57.7–90.0 %) and by negative result predictive value of 99.8 % (95 % CI 99.3–100.0 %). The optimal criterion of V2 amplitude in prognostication of postoperative vocal fold function and predictive values for selected V2 amplitude criteria are shown in Table 2.

**Table 2:** Optimal criterion of V2 amplitude in prognostication of postoperative vocal fold function and predictive values for selected V2 amplitude criteria

Optimal criterion V2				
Optimal criterion	≤189 μV			
95 % CI	0–249 μV			
Sensitivity, %	96.00			
Specificity, %	99.28			
Diagnostic accuracy for different criteria				
Criterion V2	PPV, %	95 % CI	NPV, %	95 % CI
0 μV	90.5	68.9–98.9	99.4 %	98.7–99.8
≤69 μV	82.6	61.2–95.0	99.4 %	98.7–99.8
≤100 μV	76.7	57.3–90.3	99.8 %	99.3–100.0
≤189 μV	77.4	58.5–90.6	99.9 %	99.4–100.0
≤249 μV	69.4	51.9–83.7	100.0 %	99.6–100.0

*V2* vagal stimulation following ipsilateral lobectomy, *PPV* positive predictive value, *NPV* negative predictive value

## Discussion

Intraoperative RLN neuromonitoring is an increasingly more commonly accepted method facilitating intraoperative nerve identification and additionally providing valuable information on nerve function, thus supplementing the standard of visual assessment of preservation of anatomical nerve integrity [1, 2]. Current publications state that continuous neuromonitoring may facilitate intraoperative identification of impending RLN injury, which may affect changing the surgical technique or tactics in order to prevent nerve damage [15–18]. For such an option to become valid, further studies are necessary aiming at understanding the mechanisms of RLN damage and factors affecting the predictive value of neuromonitoring, which was the subject of analysis in the present paper.

Using the definition of vagal LOS following thyroid lobe resection recommended by INMSG (V2 amplitude <100 μV), the authors noted in the presented material of 1000 RLNs at risk PPV equal to 76.7 % and NPV equal to 99.8 %. The ROC curve analysis allowed for calculating the optimal criterion in prognostication of postoperative vocal fold paresis, which was an absolute decrease of V2 amplitude V2 ≤189 μV. For this criterion, PPV was 77.4 % and NPV was 99.9 %. An additional analysis of the ROC curve illustrating the relative V2 amplitude value in relation to the initial V1 amplitude value allowed for calculating the most optimal criterion in prognostication of postoperative vocal fold paresis as a drop in the V2 amplitude below 18.4 % of the initial V1 amplitude value (or by more than 81.6 %), the criterion being characterized by PPV equal to 76.6 % and NPV equal to 99.8 %. Knowing the above predictive values, it can be calculated that the minimum V1 amplitude value which may be regarded a satisfactory initial value is 543.5 μV. The value is much higher than V1 >100 μV, which was recommended by INMSG in the 2011 guidelines [2]. In the described material, more than 95 % of patients showed V1 amplitude above this value (data not shown). This is why the predictive value of neuromonitoring in the presented material was high. However, the clinical utility of these modified criteria should be tested in a large multicenter study before any agreement is reached regarding the update of the INMSG 2011 guidelines. New publications presented by other centers also emphasize the achievable high prediction value of neuromonitoring employing standardized methodology of the test (positive—approximately 75 %, and negative—more than 99 %) [17–20]. Genther et al. analyzed results of intraoperative EMG recordings following vagal stimulation at the end of surgery (V2) in a group of 674 patients and 1000 RLNs at risk during thyroid or parathyroid surgery and compared them to the percentage of vocal fold dysfunction in postoperative fiberoptic tests. They noted that a decrease in V2 amplitude <200 μV showed positive result predictive value equal to 72.4 % and negative result predictive value equal to 99.9 % [18]. The above results are fully in accordance with the present observations. In addition, in operations employing continuous IONM, the technique which is currently advocated by an increasing number of experienced endocrine surgeons, the baseline values of EMG amplitude and latency are calibrated automatically at the beginning of surgery [15–18]. In patients in whom the initial baseline EMG amplitude is lower than or equal to 500 μV, the position of the endotracheal tube should be corrected such that maximum signal amplitudes of 500 μV or higher are obtained [18]. This issue is of paramount importance as the baseline V1 serves as a relative reference during the surgery and allows for identification of combined events which precede an intraoperative LOS. Combined events are usually defined as a 50 % or greater decrease in the EMG amplitude together with a latency increase of 10 % relative to baseline values [16]. Combined events have been shown to precede an intraoperative LOS and to normalize when the surgical manoeuver that caused stretching or pressure on the RLN was halted [15–18]. On the other hand, LOS is considered to be much more irreversible leading to RLN paresis in more than 80 % of cases [15–18]. Hence, the definition of LOS has a potential to evolve in the future as a result of ongoing debate among members of the INMSG. Actually, the outcomes of the present study support the need to validate in a large multi-institutional study also other definitions of severe LOS including decrease of V2 amplitude to less than 20 % of the initial satisfactory V1 value in prognostication of postoperative RLN function. This issue is crucial in the era of continuous IONM. Schneider at al. reported that in patients operated on with continuous IONM, there was no instance of permanent vocal fold palsy in the 1314 nerves at risk, whereas four unilateral permanent vocal fold palsies were seen in patients who had intermittent IONM (0.4 % of 965 nerves, *p* = 0.019). For continuous IONM, 63 (82 %) of 77 combined events were reversible during the operation. Thus, it seems reasonable to assume that early detection of impending nerve injury in patients operated on with continuous IONM may alert the surgeon to halt the causative manoeuver, which then may result in milder nerve injury and a better clinical outcome [18].

In practice, very high NPV of IONM, above 99.8 %, allows for safe continuation of the planned operation of bilateral thyroidectomy in case normal neuromonitoring signal is preserved following the first lobe resection, practically eliminating the possibility of bilateral RLN damage. Yet, in intraoperative LOS following one thyroid lobe resection, the decision is more difficult due to a lower PPV (approximately 75 % on average). Hence, numerous authors believe IONM to be a tool allowing for obliterating the risk of bilateral vocal cord damage providing staged thyroidectomy is planned in surgical tactics or at least limiting the scope of contralateral lobe resection in cases of LOS on the side initially operated on [10, 11, 19–21]. In the present material, of 11 patients with LOS occurring following resection of the first-excised dominant lobe, staged thyroidectomy was decided on in three cases (two recurrent goiters, one nodular goiter), while a supplementary resection of the second lobe was done 6 months after the primary procedure, having achieved restored mobility of the temporarily paretic vocal fold. In the remaining eight cases, the procedure was continued as one-stage, including three cases of thyroid cancer, where the contralateral lobe was also totally resected, four cases of toxic multinodular goiter, and one case of Graves’ disease, where the resection of the contralateral lobe was limited to subtotal resection leaving approximately 2 ml of parenchyma in the area of the Berry ligament (Dunhill procedure) to minimize the risk of bilateral RLN damage. None of the above patients demonstrated LOS during contralateral lobe resection. Similar results were also recently presented by other authors. Melin et al. retrospectively analyzed the results of 2546 thyroid procedures with IONM carried out in 2008–2010 (4012 RLNs at risk) and found early vocal fold paresis in 119 (3.0 %) patients and permanent paresis as assessed at 18 months postoperatively in 15 (0.4 %) individuals [10]. Using the criteria of intraoperative LOS defined by INMSG, the authors noted FP neuromonitoring results in 0.6 % of cases and FN in 1.2 %. In the study, neuromonitoring sensitivity was 80 %, specificity 99 %, positive result predictive value 68 %, and negative result predictive value 99 %. In 98 cases, neuromonitoring LOS occurred following resection of the first thyroid lobe; in 64 of these patients, total thyroidectomies were planned. In 24 patients, thyroidectomies were continued despite LOS following resection of the first lobe (total thyroidectomies—19 patients, and subtotal—5 patients), which led to bilateral LOS and bilateral vocal fold paresis in 4/24 (16.7 %) patients (including 1 after Dunhill procedure). However, in the remaining 40 patients, one-stage total thyroidectomies were abandoned following LOS on the first operated side. In 18 of these patients, supplementary resection of the remaining lobe was performed as stage 2 (in 16 cases following resolution of unilateral vocal fold paresis, and in 2 despite permanent unilateral vocal fold paresis); no patient demonstrated bilateral vocal fold paresis. In the remaining 22 patients, radical surgical treatment was abandoned due to lack of absolute indications or lack of patient’s consent. While assessing patient satisfaction after staged thyroidectomies, full understanding of and satisfaction from such a management model was noted in 14/18 (77.8 %) patients, while only 2/18 (11.1 %) patients professed lack of understanding and satisfaction and 2/18 (11.1 %) individuals were lost to follow-up [10]. The authors confirmed that the use of IONM was helpful in selecting patients that might benefit from staged thyroidectomies in order to prevent bilateral vocal fold paresis. In the opinion of the majority of authors, such a model is fully justified in patients with bilateral non-neoplastic thyroid disease, although some believe it acceptable in procedures performed for thyroid cancer [10, 11, 19–22]; the opinion is not shared by all experts, however [23].

Interesting data were presented by Dralle et al. in the study assessing the frequency of IONM RLN use in bilateral thyroidectomies in Germany in 2010, taking into consideration the surgeon’s awareness and willingness to change the planned surgical strategy in case of IONM loss following the first thyroid lobe resection [11]. Of 1256 German surgical wards, 1119 (89.1 %) were equipped with IONM sets. A total of 595 (53.2 %) surgical wards participated in the study; annually, they performed approximately 75 % of all thyroid procedures in Germany. IONM RLN was employed in 91.7–93.5 % thyroid operations, with 73.8 % surgeons using vagal stimulation (V2) following thyroidectomy. Almost 93.5 % of responders declared their willingness to change the planned scope of thyroid surgery in case of LOS following the first lobe resection. The extent of change ranged from abandoning contralateral resection (more commonly in benign goiter, especially without laryngeal compression) to limiting thyroidectomy to subtotal resection of the contralateral lobe (Dunhill procedure) to minimize the risk of postoperative bilateral vocal fold paresis.

The decision to employ the model of staged thyroidectomy in clinical practice requires discussing the issue with the patient prior to planned surgery [10, 11, 19]. As confirmed by our experience and the published data, patients most often understandingly accept such a solution, especially when qualified for thyroid reoperation due to recurrent goiter when the risk of RLN damage is higher than in primary operations [8].

Despite a prospective design, the current study has several limitations. Not all eligible patients were included, as the employment of IONM in Poland is not reimbursed by the Polish National Health Fund which is the main reason for using this technique on select patients depending on the individual surgeon’s preferences and availability of the equipment. The operations were performed not by one, but two (MS, MB) surgeons. However, in order to minimize the risk of any bias, all the surgeons involved in this study had a comparable background experience both in thyroid surgery and utilization of IONM during thyroidectomy. Postoperative laryngoscopy was not performed immediately after surgery in the recovery room, but at postoperative day 1, which in theory might have led to underestimation of the prevalence of short-lasting transient nerve injuries [24].

In the nearest future, we should expect further popularization of neuromonitoring in thyroid and parathyroid surgery, since its cost accounts for only 5–7 % of total cost of thyroidectomy and the value of this method in preventing bilateral RLN damage—a complication being the most common cause of claim awarding in civil lawsuits—is presently well-documented [25, 26].

## Conclusions

Adherence to the standardized protocol recommended by INMSG allows for optimizing predictive values of intraoperative neural monitoring in prognostication of postoperative RLN function. Negative predictive value of the method equal to 99.8 % allows for safe continuation of planned bilateral thyroid surgery in case of intact neural monitoring signal after unilateral thyroid lobectomy. Positive predictive value of the method equal to 76.7 % makes it a rationale for staged planned total thyroidectomy in case of LOS after unilateral thyroid lobectomy in order to abolish the risk of bilateral vocal fold paresis. Any changes in the cutoff values for the definition of LOS only marginally improve the clinical benefit (PPV and NPV) of IONM and need to be carefully assessed in multicenter studies.
